# A Patient with Acute Necrotizing Fasciitis after a Total Knee Replacement: A Case Report

**DOI:** 10.3390/diagnostics13061125

**Published:** 2023-03-16

**Authors:** Shu-Hao Chang, Ching-Chuan Jiang, Tom J. Liu, Yu-Feng Kuo, Ping-Chun Yeh

**Affiliations:** 1 Department of Orthopedics, Fu Jen Catholic University Hospital, Fu Jen Catholic University, No. 69, Guizi Rd., Taishan Dist., New Taipei City 24352, Taiwan; 2 School of Medicine, College of Medicine, Fu Jen Catholic University, No. 510, Zhongzheng Rd., Xinzhuang Dist., New Taipei City 24205, Taiwan; 3 Division of Plastic Surgery, Department of Surgery, Fu Jen Catholic University Hospital, Fu Jen Catholic University, No. 69, Guizi Rd., Taishan Dist., New Taipei City 24352, Taiwan

**Keywords:** necrotizing fasciitis, total knee replacement, fungal infection, Enterococcus faecalis, gastrointestinal

## Abstract

Necrotizing fasciitis is a relatively rare and serious fatal soft-tissue infection that is characterized by a rapidly spreading bacterial infection located in the subcutaneous tissues. We report a 59-year-old man who was diagnosed with acute necrotizing fasciitis, following a primary total knee replacement. He received primary total knee replacement that was uneventful and smooth intraoperatively. An immediate high fever was reported in the next few days, with several complications, confirming a diagnosis of necrotizing fasciitis. The most effective treatment for this disease is a rapid primary diagnosis and surgical debridement. Gold standard treatment includes intravenous therapy, such as antibiotics, surgical debridement, and intensive care. As a result of possible GI complications that triggered necrotizing fasciitis, the patient underwent flap reconstruction. This report’s aim is to review the comprehensive treatment, management, and experience of necrotizing fasciitis, highlighting the roles with a multidisciplinary care team for improving the condition of this patient.

Figure 1A shows the initial radiography revealing bilateral knee Kellgren–Lawrence grade IV osteoarthritic change, with Figure 1B showing the medial joint space narrowing mostly on the right knee. Figure 1C,D shows the successful total knee replacement (TKR) on the right knee. A 59-year-old man has a history of hypertension for over ten years and receives regular medication control for hypertriglyceridemia and depression. His surgical history includes laparotomy surgery for perforated peptic ulcers and an appendectomy. He suffered from bilateral knee pain for years, especially in his right knee. The knee pain aggravated while walking and climbing up the stairs, and therefore, he was seeking medical help. Once TKR was successful, two days after his operation, a sudden fever of 40.5 Celsius degrees developed, accompanied by nausea, vomiting, and abdominal distention. Laboratory data revealed leukocytosis (white blood cells: 12.81/uL), elevated C-reactive protein (41.22 mg/dL), and elevated procalcitonin (5.20 ng/mL). Further workup was arranged for identifying the possible infection, focusing on the gastrointestinal (GI) tract. His esophagogastroduodenoscopy examination only showed reflux esophagitis and superficial gastritis, and his colonoscopy reported no positive findings. The abdominal ultrasound also did not identify any abdominal site of infection. However, persistent fever was still noted. The antibiotics were customized by the infection specialist. Necrotizing fasciitis (NF) is a severe infection of the subcutaneous tissue, characterized by the necrosis of the subcutaneous tissues and fascia, and is usually results from a group A beta-hemolytic streptococcal infection or polymicrobial synergistic infection [1]. Mortality from this infection is significant if left untreated or if treatment is delayed. Early diagnosis and definitive management are paramount because of the rapidly progressing nature of the infection [2,3,4,5]. The incidence of this disease has increased about five-fold during the last decade, which can be partly due to an increase in the number of immune-deficient patients, and may even be due to frequent reports during recent years. Mortality rates range from 30% to 90%, according to the recently published book [6]. Gastrointestinal symptoms are also one of the possible predictors of severe outcomes of invasive group A streptococcal infections, as reported in a 2010 study [7]. As a result of the high mortality rate in patients suffering from this disease and the lack of adequate research in this field, we wanted to record the complications and symptoms that have occurred along with several examinations and tests that we have analyzed up until the removal of implements, and the acceptation of flap reconstruction.

Figure 2 shows the left knee surgical wound developed oozing with turbidity and left thigh-to-knee redness, and swelling deteriorated one week postoperative. Left knee septic arthrotomy with debride and removal of the prosthesis with cement spacer implantation were done on 8th day post-operation. The patient was later transferred to the intensive care unit. The wound culture yielded enterococcus faecalis, the antibiotic with ampicillin was added, in addition to keeping the antibiotic therapy with daptomycin and doripenem. Due to persistent yellowish slough and turbid bloody fluid from the left knee wound, debridement, and regional fasciectomy were repeated two weeks postoperatively. We then shifted the left knee wound management to negative-pressure wound therapy. Serial regional fasciotomies were done for removing necrotic tissue. Figure 2 shows the development of the left leg infection throughout the stay in the hospital. The patient was kept on the antibiotic therapy with ceftazidime in addition to vancomycin to control the infection. The wound culture taken in subsequent debridement revealed Enterococcus faecalis, while the sputum culture revealed Candida Albicans. Antibiotics were adjusted accordingly. Free flap reconstruction was performed one month after the TKR procedure, and the skin flap was taken successfully two weeks later. To our understanding, the previous case report that was published with a similar case was in 2011 [1], with one older case report in 2003 [8]. Table 1 shows the comparison between their cases and managements, with this case report. Other related cases with TKR that were researched were two other case reports of total hip replacement that were only reported in 2007 [9] and 2000 [5]. While Roth et al. reported that the cause for the 71-year-old female patient was an infection with a multi-resistant Staphylococcus aureus, Steckel et al. reported that there was no bacterial infection found in the microbiological examination for the 65-year-old female patient. However, after their histological examination, they did find numerous Gram-positive cocci that could be seen in the necrotic areas. In our case report, we found Enterococcus faecalis (Gram-positive bacilli). From this experience, we suspect that patients with GI disease in the past are more likely to acquire opportunistic infection, caused by the normal flora in GI tract. Enteococcal bactermia (EB) may play a key role in acute surgical wound infection, with rapid progression in our presented case. Enterococcus faecalis and Enterococcus faecium are members of the normal flora of the gastrointestinal tract, but are also typical opportunistic pathogens. In a review by Turco et al., some patients with poor immunity often had asymptomatic EB [10]. In this case, even when the pathogen enterococcus was a normal intestinal bacterium, when the surgery was performed, it very likely caused acute and rapid wound infection complications. As the high mortality rates of EB pose an increasing challenge for clinicians, key risk factors, such as the patient’s baseline condition, underlying comorbidities, the presence of complicated bacteraemia, antimicrobial resistance, the need for intensive care admission, and the appropriateness of antibiotic therapy, are all heavily influenced for appropriate treatment management [5]. Similarly to our case, the patient underwent laparotomy for peptic ulcers five years ago and this may be a reason why clostridium is a pathogen in this opportunistic infection. In a brief report in 2000, of the 15 patients that were diagnosed with NF missed at the initial evaluation, 47% of them had gastroenteric symptoms, such as nausea, vomiting, and diarrhea [11]. In an epidemiological analysis study in 2010, they reported that GI symptoms were significantly associated with NF with group A streptococcal pathogens [7]. They claimed that streptococcal exotoxins may be the common cause of NF and GI symptoms. These exotoxins arbitrate their damage by increasing cell membrane permeability. Due to three possible permeability changes (trans-membrane pore formation; altering lip composition of the membrane; or the detergent-like activity of the toxin), this altered permeability leads to cell death by disrupting cell function or lysis, or influx of ions [7]. With exposure to Streptococcus pyogenes (group A Streptococcus), these bacteria could gain access to the systemic circulation by destroying the cells that compromise the barrier. This loss of cells would expose the host to invasion by the bacteria, therefore, this is the possible mechanism by which diarrhea and/or vomiting GI symptoms are correlated with the subsequent observation of NF [7]. This could be explained by GI exposure (a simple columnar epithelium) being more likely to lead to systemic translocation than pharyngeal exposure (a stratified squamous epithelium). Therefore, if patients have early signs of GI complaints, such as diarrhea and/or vomiting in the initial course of the examination, physicians should also take note of the possible early stages of invasive NF infection. Treatment of NF is surgery, and the earlier the surgery is, the better the outcome. From the two previous case reports that were compared to this case report, amputation was necessary, while our case utilized free flap reconstruction. In a recent surgical management of NF in the lower extremities that collected 62 patients in a 10-year retrospective review, 42 patients underwent amputation [12]. Amputation is considered the last option, assessed according to the wound care and by specialists. Early decision for surgery may eliminate the need for amputation and may help minimize tissue loss. Most amputations are performed as a consequence of a failed arthroplasty in the lower extremities, to treat severe infection, pain, or massive bone loss. In conclusion, the clinical relevance of this case report is that NF should be addressed early and aggressively. Clinical judgment and a high index of suspicion for NF will ultimately expedite recognition. Such severe postoperative infection should be kept in mind, especially in patients with gastrointestinal, mental, or psychiatric disorders that weaken immunity. Possible contributing factors to infection need to be eliminated before elective surgeries.

## Figures and Tables

**Figure 1:**
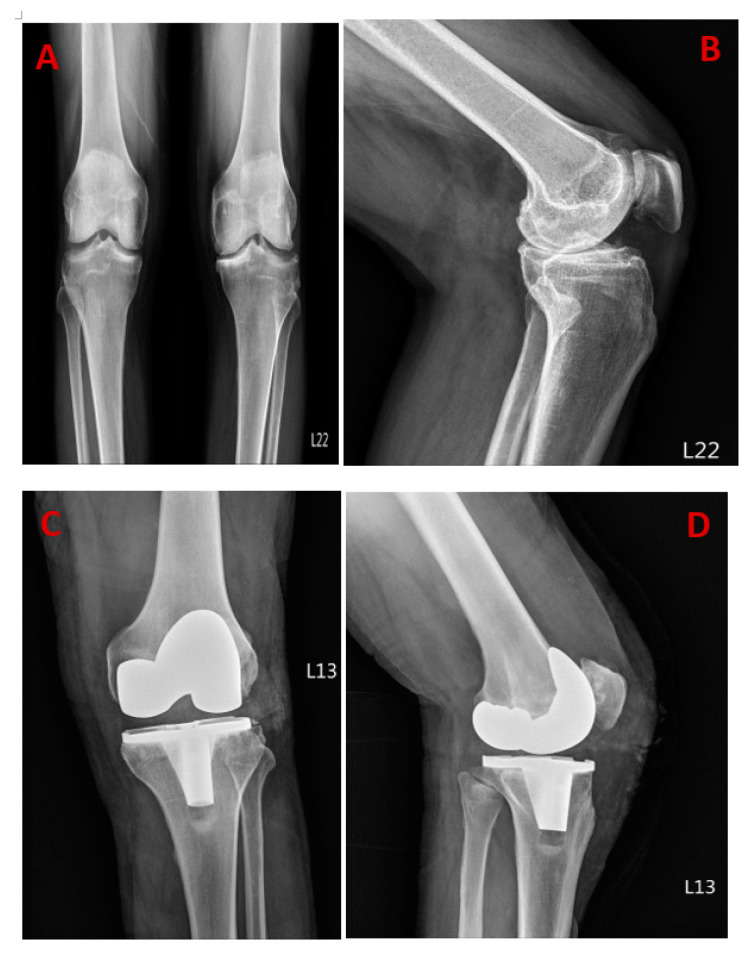
Initial radiography images before total knee replacement (**A**,**B**) with successful post-surgery radiography (**C**,**D**).

**Figure 2:**
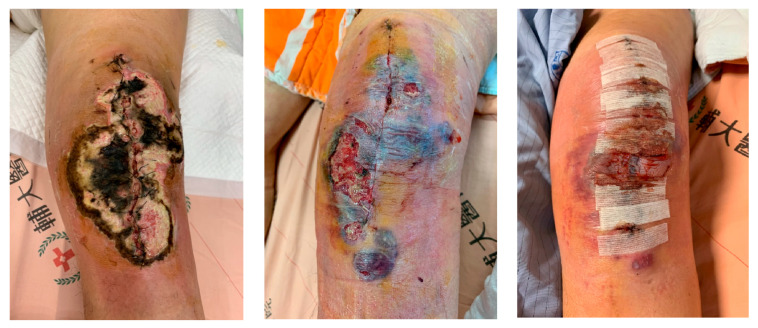
Acute necrotizing fasciitis infection happening one week postoperative.

**Table 1:** Comparison between this case report and two previous similar case reports.

Case Report	Baseline Characteristics	History	Main Compliance for Visit/Surgery	Infection after Surgery	Treatment and Management
Roth et al. 2003 [8]	71-year-old, F	Diabetes Type II, arterial hypertonia	Bilateral knee pain, more at the right knee, underwent TKR	Septic infection with a multi-resistant Staphylococcus aureus	Systemic and local application of vancomycin showed improvements; however, amputation of the primarily infected leg was done in the end.
Steckel et al. 2011 [1]	65-year-old, F	Embolism, a deep venous thrombosis, and a factor V Leiden	Osteoarthritis on left knee joint, underwent TKR	Abscesses, ulcerations, necrosis, and a high-grade osteomyelitis	Antibiotic therapy was administered from the initial Tazobactam/Piperacillin to Clindamycin/Linezolid. Above-the-knee amputation was done in the end.
Our case report	59-year-old, M	Hypertension, laparotomy surgery for peptic ulcers, and an appendectomy	Bilateral knee pain, more at the right knee, underwent TKR	Acute necrotizing fasciitis, Enterococcus bacteremia	Antibiotic therapy with ceftazidime/vancomycin was administered. Free flap reconstruction was done in the end.

## Data Availability

Not applicable.
